# Impact of Ammonia Exposure on the Enteric Nervous System in the Ileum of Pigs From Birth

**DOI:** 10.1111/nmo.70156

**Published:** 2025-09-12

**Authors:** Valentina Rafaela Herrera Millar, Mirko Sergio, Katarzyna Palus, Giorgio Mirra, Chiara Cialini, Margherita Pallaoro, Lucia Aidos, Laura Mangiavini, Chiara Bazzocchi, Eleonora Buoio, Annamaria Costa, Silvia Clotilde Bianca Modina, Alessia Di Giancamillo

**Affiliations:** ^1^ Department of Biomedical Sciences for Health University of Milan Milan Italy; ^2^ Department of Veterinary Medicine and Animal Science University of Milan Lodi Italy; ^3^ Department of Clinical Physiology University of Warmia and Mazury Olsztyn Poland; ^4^ Technical University of Denmark National Institute of Aquatic Resources Kongens Lyngby Denmark; ^5^ IRCCS Ospedale Galeazzi – Sant'ambrogio Milan Italy

**Keywords:** ammonia, ENS, gut, neuronal plasticity, pig

## Abstract

**Background:**

Best Available Techniques have been introduced in the EU to counteract pollution related to intensive farming and its effect on the health of animals and workers. It is known that high levels of ammonia (NH_3_) worsen the productive performance of animals, but the exact mechanism of interaction with the intestine is still unknown. Therefore, this study aimed to investigate the effect of NH_3_ on the gut of pigs exposed to different levels since birth.

**Methods:**

Two farms with different manure removal systems were considered (Recirculation and Vacuum), where two different NH_3_ levels were detected: high and low ammonia (recirculating slurry system, RS, and vacuum slurry system, VS, respectively). The entire production cycle was considered, and a fecal score and microbiological analyses of the feces were performed. After slaughtering, the ileum of 12 animals was sampled to evaluate ileum morphology and the expression of some neurotransmitters.

**Key Results:**

No differences were found in Peyer's patches morphology. The mucus layer thickness was higher, and the acidic mucins were lower in the RS animals. Results revealed changes in the number and area of neuronal and glial cells, and an upregulation of choline acetyltransferase (*ChAT*) and galanin (*GAL*) genes was observed.

**Conclusions and Inferences:**

The alteration of the Enteric Nervous System (ENS) highlighted a connection between high levels of environmental NH_3_ and neuroplasticity. Furthermore, the upregulation of *ChAT* and *GAL* genes suggests a key role in visceral pain, creating a link between peristalsis and chronic diarrhea observed in healthy pigs. Lastly, these findings are important for both animal health and human workplace safety.

## Graphical abstract

Created in BioRender. Pallaoro, M. (2025) https://BioRender.com/706fdhr.

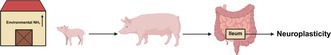

**Summary**

- NH_3_ is an environmental threat to both farm animals and workers.
- NH_3_ alters neuroplasticity of the Enteric Nervous System.
- Farmed pigs may represent a good model to study the effect of air pollutants on intestinal health.

## 1 Introduction

Intensive livestock farming constitutes more than 70% of the total emissions of NH_3_, among other gases [1]. To counteract pollution, the EU introduced the Best Available Techniques in the ILF BREF (Best Available Techniques (BAT) Reference Document for the Intensive Rearing of Poultry or Pigs—Publications Office of the EU, n.d.), suggesting sustainable changes to facilities, buildings, and management systems [2, 3]. Among the many advantages, there are the improvement of animal health and the guarantee of a safe environment for workers: high levels of NH_3_ in the working environment cause respiratory and cardiovascular diseases [4, 5, 6]. Manure management on farms is one of the main factors affecting the levels of NH_3_ in the environment [7].

Some studies have shown a worsening in pig performance in correspondence with high levels of NH_3_ [8, 9] and such deterioration could be related to a consequent reduction in the amount of food eaten or to a lower efficiency in the use of nutrients due to a state of general distress or disease [10]. Moreover, the pig farm environment could represent a risk factor for gastrointestinal disorders in animals. However, the mechanisms through which the environment and its possible pollutants interact with the intestine are poorly understood. Recently, Zhang et al. [11] observed that NH_3_ is neurotoxic, increasing the apoptosis of nerve cells in broiler chickens. Furthermore, Li et al. [12] observed a change in jejunal flora in fattening pigs exposed to ambient NH_3_, associated with neutrophil activation, resulting in intestinal inflammation. Finally, recent research has highlighted the importance of the interaction between the Enteric Nervous System (ENS) and inflammation to maintain intestinal homeostasis [13]. Neurons and glial cells are part of the ENS located in the ganglia of the intestinal wall [14, 15]. From a structural point of view, neurons are usually surrounded by many enteric glial cells, non‐neuronal and supporting cells, and have different neurochemical coding and functional roles based on location [16]. Generally, the submucosal plexus (SP) governs the movement of water and electrolytes across the intestinal wall [17], while the myenteric plexus (MP) coordinates the contractility of the circular and longitudinal muscle cells to produce peristalsis [18]. Glial cells surround neurons [19] and have a unique characteristic which is the abundance of Glial Fibrillary Acidic Protein (GFAP) in their cytoplasm [20]. Enteric glia interacts with enterocytes, enteroendocrine, and immune cells, which are emerging in regulating intestinal functions and inflammation. The ENS, thanks to its complex neuronal network, interacts with the immune centers located in the small intestine, such as the Gut‐Associated Lymphoid Tissue (GALT), modulating immune responses through neurochemical signals that influence the activity of immune cells and surveillance. The GALT in the ileum consists of isolated or aggregated lymphoid follicles forming Peyer's Patches (PPs), located in the lamina propria of the distal portion. PPs are mentioned as the immune sensors of the intestine due to their ability to transport luminal antigens and bacteria [21]. The GALT is rich in innervation, which is derived from the ENS, and with which it reciprocally exchanges information [22]. It has been observed that the nerves and fibers present in the PPs can contribute to the dialogue between the nervous system and the immune system (neuroimmune crosstalk). This occurs at antigen sampling sites, where the immune system recognizes and analyzes foreign substances [23]. Furthermore, nerves can integrate and coordinate information from different sampling sites, contributing to a more effective and synchronized immune response [24, 25]. The enteric ganglia are located at the base and corona of the PPs follicles as well as in the interfollicular regions. ENS has a significant ability to adapt to microenvironmental influences throughout life, through a mechanism called “neuronal plasticity.” Major features of neuroplasticity encompass structural abnormalities ranging from nerve re‐arrangement (e.g., hypertrophy and hyperplasia) to degeneration and loss of enteric ganglion cells, altered synthesis, content, and release of neurotransmitters as well as up‐ or downregulation of receptor systems, or gastrointestinal dysfunction characterized by sensory‐motor and secretory impairment of the gut. Enteric neurons can interact with smooth muscles, epithelium, or immune cells, through the secretion of a wide range of neuropeptides and neurotransmitters [26]. Among these, the neurotransmitter acetylcholine plays an excitatory role and is synthesized by the enzyme Choline O‐Acetyltransferase [27]. The Nitric Oxide Synthase (NOS) belonging to the family of enzymes catalyzing nitric oxide production is an important inhibitory marker for enteric neuronal transmission [28, 29]. In mammals, NOS exists in three forms, and among them, inducible NOS (iNOS) is a key mediator of immune activation and inflammation [30]. Moreover, the neuropeptide Galanin (*GAL*) is receiving a lot of interest as it is involved in the perception of intestinal pain [31]. This peptide plays a key role in chronic pain conditions and its anti‐nociceptive action. A better comprehension of ENS plasticity during inflammation could be instrumental in developing new therapeutic options for inflammatory enteric neuropathies. This study aims to understand the effects of exposure of pigs to NH_3_ in the early phases of life on the development of the ENS and its impact on adult life until slaughter. Since farm workers are also exposed to NH_3_, this study may give important information on the security of the working environment.

## 2 Materials & Methods

### 2.1 Experimental Design

#### 2.1.1 Farm Environment

**FIGURE 1:**
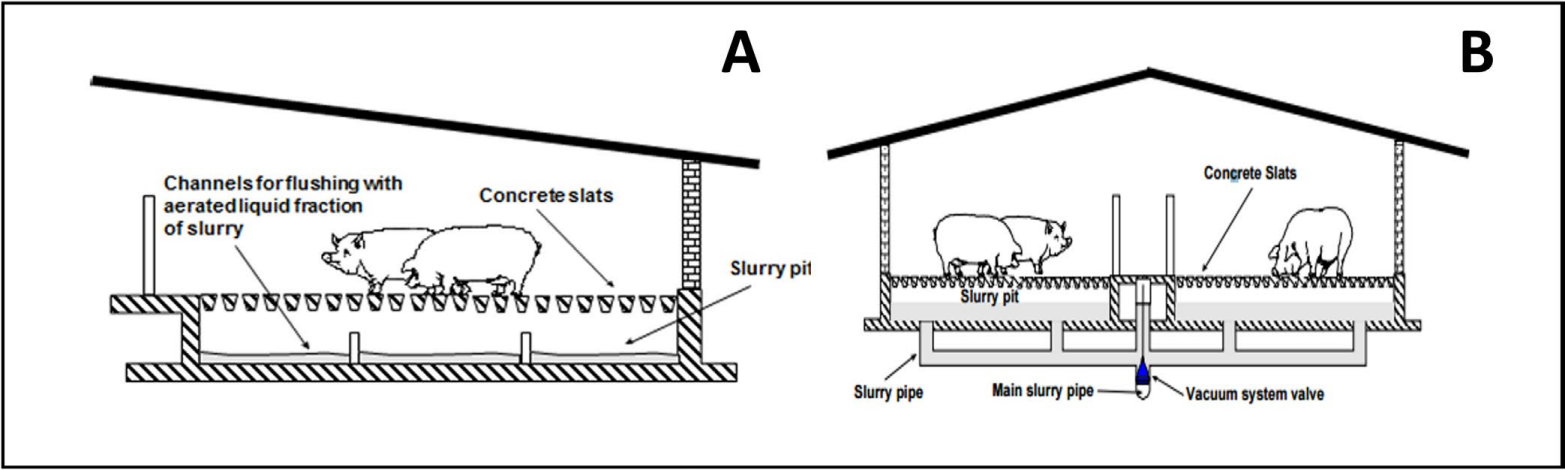
(A) Recirculation system (RS) rooms, with a fully slatted floor and underneath channels with a permanent slurry layer for manure recirculation; (B) Vacuum system (VS) rooms with a fully slatted floor and the vacuum system under the pit. In: Best Available Techniques (BAT) Reference Document for the Intensive Rearing of Poultry or Pigs https://data.europa.eu/doi/10.2760/020485.

Two different farming systems were considered to assess the influence of the farming environment on the intestinal health of the pigs. Both farms are classified as intensive, with more than 2000 pigs with a weight higher than 30 kg, according to the Directive on Integrated Prevention Pollution and Control (IPPC, 1996) indications, rearing fattening pigs to produce Parma Ham. In the two farms, “Best Available Techniques” (BAT), able to mitigate NH_3_ concentration and emission through the building, plants, and management type are adopted [32]. Briefly, the first farm presented a recirculation system (RS) under the floor, while the second presented a vacuum system (VS) to remove slurry (Figure 1).

In the RS farm, farrowing rooms housed eight sows, and in the VS farms, 30 sows. In the two farms, after 28 days with the mother, the piglets were moved to the weaning unit (around 70 days of), then to the first fattening unit (90 days), and the second and last fattening unit (another 90 days). Then, at the age of around 9 months and at a live weight of around 150 kg, pigs were slaughtered. In each unit, accommodation, flooring, and space requirements were guaranteed for animals, according to EU welfare rules (Council Directive 2001/93/EC).

In both farms, the NH_3_ concentration was measured twice a month for two months before the beginning of the trial, as a preliminary trial to detect potential differences in ammonia concentration between the two farms during farrowing. During the observation periods, which included one month for the farrowing rooms and three months for the fattening rooms, NH_3_ was measured once an hour for eight hours, one day a week, from 9 a.m. to 5 p.m., for a total of nine measurements per point per room. Measurement was performed with a detector (GasBAdge Pro NH_3_, Industrial Scientific, Pittsburgh, PA, USA, accuracy ±5%) in each room at different points to obtain information on the air quality in the pens and the aisle. Sampling was carried out at 1 m of height, a compromise between animal and men's height. The farms, mechanically ventilated, were already set up to measure temperature, relative humidity, and ventilation rate in the rearing rooms, automatically controlled by the Fancom FMS‐type ventilation system, which worked according to the temperature requirements of the animals.

### 2.2 Animals Management

The groups consisted of newborn piglets on both farms, housed in the farrowing rooms. At weaning, piglets were transferred to the fattening rooms until slaughter. In compliance with the 3Rs principle (Replace, Reduce, Refine), no animals were sacrificed for experimental purposes. Pigs were reared for commercial purposes, and at the slaughterhouse, ileum samples were collected. The Animal Welfare Organisms of the University of Milan were informed about the collection of waste tissues, such as the ileum, as required by national legislation. Animals did not undergo pharmacological treatments before being slaughtered. The farm veterinarian performed animal health, welfare, and fecal scores as an internal *Hazard Analysis and Critical Control Points* (HACCP) control.

### 2.3 Fecal Score and Microbiological Analysis

Fecal consistency was scored daily as follows: formed/normal, 0; soft/wet cement consistency, 1; runny/watery, 2; mucoid diarrhea, 3; bloody diarrhea, 4. Colon fecal samples were collected at the slaughterhouse, and the bacterial count was performed for *Streptococcus* spp., *Enterobacteriaceae* spp. (*Escherichia* spp., *Salmonella* spp.) and *Lactobacillus* spp. The microbial populations were log‐transformed, and data were expressed as log_10_ Colony‐Forming Units (CFU)/grams of digesta.

### 2.4 Ileum Morphology

Six ileum samples were collected at the slaughterhouse from pigs randomly selected from each farm. They were immediately washed in phosphate‐buffered saline (PBS) solution and stored in 10% neutral buffered formalin for 24 h. Samples were washed for three hours in running water and kept in 70% alcohol overnight (O.N.) at 4°C, dehydrated to a growing scale of alcohol (95% and 2 × 100%) at room temperature (R.T.), clarified to xylene, and paraffin‐embedded.

#### 2.4.1 Morpho‐Functional Analyses

##### 2.4.1.1 Histology

Microtome sections (5 μm thick) were stained with hematoxylin–eosin (HE) to establish structural details. The images were acquired with a light microscope (Optika B‐510BF, Olympus, Opera Zerbo, Milan, Italy) equipped with a digital camera, and for each sample, six measurements were made to evaluate the percentage of area occupied by the *corona*, by the *cortex* (around the *medulla*), and by the *medulla* (the innermost area) of the PPs compared to the total area [33].

##### 2.4.1.2 Histochemistry

Other sections of the ileum were stained to determine the mucin profile. For this purpose, the following histochemistry stain was applied: Alcian Blue 8GX pH 2.5‐Periodic Acid Schiff (AB‐PAS) sequence, revealing neutral (PAS‐reactive, purple‐stained) and acid (AB‐reactive, azure‐stained) glycoconjugates [34]. The images were acquired with a light microscope (Optika B‐510BF, Olympus, Opera Zerbo, Milan, Italy), equipped with a digital camera, and the following parameters were determined: the thickness of the mucous layer (mean of 15 measurements per sample) [14], the area occupied by the goblet cells relative to the area of the epithelium (mean of the ratio of 3 figures per sample), and the percentage of acidic and neutral cells (at least 100 cells counted per sample and calculated the relative ratio to the number of total cells) according to the literature [35, 36].

##### 2.4.1.3 Double Immunofluorescence

On other ileum sections, double immunofluorescence was performed to characterize the enteric nervous system ganglia, both myenteric and submucosal, according to our previous study [14]. After rehydrating the tissue sections, unmasking was performed for antigen retrieval, using heat‐induced microwaving in citrate buffer at pH 6 for five minutes with the microwaves at 500 W, followed by cooling twice. The sections were washed three times in phosphate‐buffered saline (PBS, pH 7.4) and treated with the Avidin‐Biotin blocking kit solution (Vector Laboratories Inc., Burlingame, CA, USA). Afterward, the sections were incubated with the first‐step primary antiserum 1:200 anti‐GFAP (Glial fibrillary acidic protein rabbit polyclonal, Dakocytomation, N1506) for 24 h at room temperature. After washing in PBS, the sections were incubated with 10 g/mL of a solution of goat biotinylated anti‐rabbit IgG (Vector Laboratories Inc.) in PBS for two hours at room temperature. Then, the sections were treated with 10 g/mL Fluorescein–Avidin D (Vector Laboratories Inc., Newark, NJ, USA) in 0.1 M NaHCO_3_ at pH 8.5 and 0.15 M NaCl for 2 h at room temperature after rinsing with PBS. The second step of the double immunofluorescence procedure consisted of treating the sections with primary antiserum 1:200 anti‐PGP9.5 (Anti‐Protein Gene Product 9.5 monoclonal antibody 31A3, Cat. No. MA1‐83428 Invitrogen, MA, USA) for 24 h at room temperature. The sections were washed in PBS for 10 min and incubated with 10 g/mL goat biotinylated anti‐mouse IgG (Vector Laboratories Inc., Newark, NJ, USA) for 2 h at room temperature. Afterward, the sections were rinsed twice in PBS and treated with 10 g/mL Rhodamine–Avidin D (Vector Laboratories Inc. Newark, NJ, USA) in 0.1 M NaHCO_3_ at pH 8.5 with 0.15 M NaCl for 2 h at 18°C–20°C. Lastly, tissue sections were fixed in Vectashield Mounting Medium with DAPI (SKU H‐1200‐10; Vector Laboratories Inc., Newark, NJ, USA) and examined using a confocal laser scanning microscope (FluoView FV300; Olympus). Argon/Helio–neon–green lasers with excitation and barrier filters set for rhodamine were used to excite the immuno‐fluo‐reactive structures. Images involving overlapping fluorescence were obtained by sequentially acquiring the image cut of each laser excitation. The omission of the primary antibody during the first incubation step guaranteed the absence of cross‐reactivity with the secondary antibody. Images containing superimposition of fluorescence were obtained by sequentially acquiring the image slice of each laser excitation or channel.

Image analysis was performed using ImageJ software (National Institute of Health, Bethesda, MD, USA). The considered total surface for all measurements was the intestine cross‐sectional area. Each ganglion has been recognized as a cluster of neuroglial cells immunopositive for the respective antibodies, as reported by Sasselli et al. [37]. Immunoreactive neurons per ganglion were counted in the ENS of the ileum in the submucosal plexus and myenteric plexus, according to Di Giancamillo et al. [16]. The total number of ganglia was evaluated on the considered area and expressed as total number/mm^2^ [38]. Neuron and glial density were evaluated as the immunoreactive area on the total considered area and expressed as a percentage [39]. To assess neuronal‐glial interactions, the authors quantified the ratio of neural density/glial density (PGP9.5/GFAP ratio) [40].

### 2.5 Neurotransmitters Gene Expression

#### 2.5.1 RNA Extraction and cDNA Synthesis

Total RNA was extracted from each sample using the miRNeasy FFPE Kit (Qiagen, Hilden, Germany), eluted in a final volume of 30 μL of RNase‐free water, and quantified using a NanoDrop 8000 spectrophotometer (Thermo Scientific, USA). Deparaffinization using xylene was performed to remove paraffin from the samples and enable their exposure to proteinase K, following the manufacturer's protocol. Five hundred nanograms of RNA were retro‐transcribed to cDNA using the Quantitect Reverse Transcription Kit (Qiagen, Hilden, Germany) according to the manufacturer's instructions. The removal of any potential genomic DNA contamination was ensured by performing a double treatment with the DNase enzyme. cDNAs were stored at −80°C until subsequent use.

#### 2.5.2 Gene Expression Profiles

The expression of the genes coding for Choline acetyltransferase (*ChAT*), galanin (*GAL*), and Inducible nitric oxide synthase (*iNOS*) was analyzed. *β‐actin* was used as a reference gene. Primer sequences, annealing temperatures, and the amplification size of each fragment are described in Table 1. Quantitative Real‐Time PCRs were performed using an iQ5 Real‐Time PCR instrument (Bio‐Rad, CA, USA) and Universal SYBR Green Supermix (Bio‐Rad, CA, USA) as a fluorescent molecule. The amplification conditions were 250 nM (final concentration) of forward and reverse primers for all genes. The thermal profile was 95°C for three minutes, followed by 40 cycles of 95°C for 20 s and 59°C for 60 s for *β‐actin*, *ChAT*, and *GAL* genes, while 95°C for three minutes and 40 cycles of 95°C for 15 s, 55°C for 15 s, and 72°C for 30 s for the *iNOS* gene. A melting profile was included. The efficiency of Real‐Time PCR was defined by the standard curve method. Cycle threshold (Ct) values were determined for each sample and normalized using the *β‐actin* gene as an endogenous control. The relative gene expressions of samples collected from control and treated groups were calculated using the ΔΔCt method and were compared to one of the control samples, considered as a calibrator.

**TABLE 1:** Primer sequences used in real‐time PCR showing the forward and reverse sequences, the annealing temperatures, and the amplicon lengths.

Gene	Primers	*T*_*m*_	Size (bp)	References
*ChAT*	F: GCTAGCCTTCTACAGGCTCC R: AGTGGCCGATCGAATGTTGT	61.4°C 57.3°C	103	[41]
*GAL*	F: TGGGCCACATGCCATCGACA R: CGGCCTGGCTTCGTCTTCGG	61.4°C 65.5°C	94	[41]
*iNOS*	F: CAACAATGGCAACATCAGG R: CATCAGGCATCTGGTAGC	54.5°C 56°C	119	[42]
*β‐actine*	F: GACATCCGCAAGGACCTCTA R: ACACGGAGTACTTGCGCTCT	59.3°C 59.3°C	157	[43]

### 2.6 Statistical Analysis

Data related to NH_3_ concentrations and environmental parameters were submitted to analysis of variance (Proc GLM) of the SAS 9.4 statistical package, to evaluate the effect of the farm, intended as the manure removal system, considering the environmental parameters (T and RH) and ventilation rate as coeffects. Normality tests were performed (Shapiro–Wilk and Kolmogorov–Smirnov), and when data were normally distributed, the parametric test was executed (unpaired *T*‐test); otherwise, the nonparametric test was performed (Mann–Whitney). Data were considered significantly different when *p*‐value (*p*) was lower than 0.05. Data were illustrated in a column graph representing the mean plus the standard error of the mean (SEM).

## 3 Results

### 3.1 Farm Environment

**FIGURE 2:**
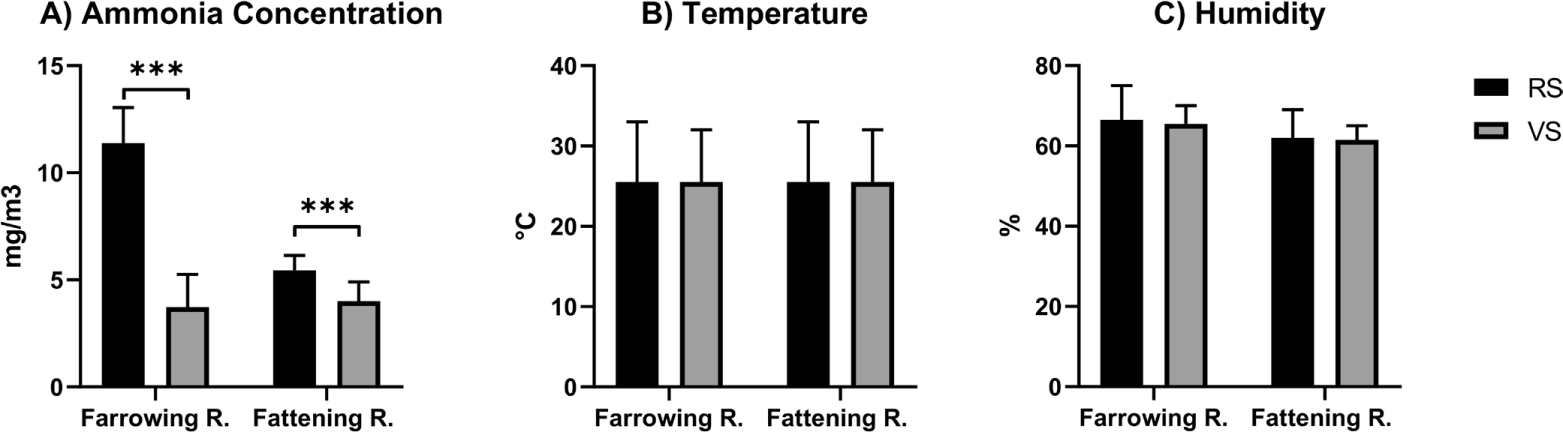
(A) Ammonia concentration, (B) Temperature, (C) Humidity in the RS and VS farm. Results are expressed as mean ± SEM. ***p < 0.001.

The mean values of NH_3_ concentrations were significantly different between the two farms. The first farm with a recirculation system had significantly higher levels of ammonia than those with a vacuum system. For this reason, from now on, the farms will be indicated as high levels of ammonia (RS) and low levels of ammonia (VS) to be more quickly understandable. Specifically, the RS had higher NH_3_ levels than VS, both in the farrowing room (*p* < 0.001, Figure 2A) and in the finishing room (*p* < 0.001, Figure 2A). In the farrowing rooms, NH_3_ levels were even three times higher in the farm RS. The temperature and relative humidity (RH%) in the farrowing and fattening rooms were also recorded, but no significant differences were observed between the two farms (Figure 2B,C, respectively). The ventilation rate was similar in the two farms, 4920 m^3^/h in RS and 5010 m^3^/h in VS for farrowing rooms, 30,210 m^3^/h in RS, and 32,000 m^3^/h in VS for fattening rooms.

### 3.2 Fecal Score and Microbiological Analysis

The mean range of fecal score has been performed by the veterinarian; consequently, only the mean has been presented. All the animals reached the estimated healthy slaughter weight (around 160 kg), and they showed no signs of illness, as indicated by the farm veterinarian. The mean fecal score was 4.5 and 2.7 for RS and VS farms, respectively. No differences between the two farms were observed concerning microbiological analysis (Table 2).

**TABLE 2:** Microbiological analyses.

	RS farm	VS farm
Total bacterial count	7.21 ± 0.40	7.00 ± 0.01
*Enterobacteriaceae* spp.	6.96 ± 0.42	7.41 ± 0.50
*Streptococcus* spp.	6.57 ± 0.39	5.91 ± 0.53
*Lactobacillus* spp.	6.63 ± 0.65	6.52 ± 0.71

*Note:* Values are expressed as log10 colony‐forming units/g of digesta.

### 3.3 Ileum Morphology

#### 3.3.1 Histology

**FIGURE 3:**
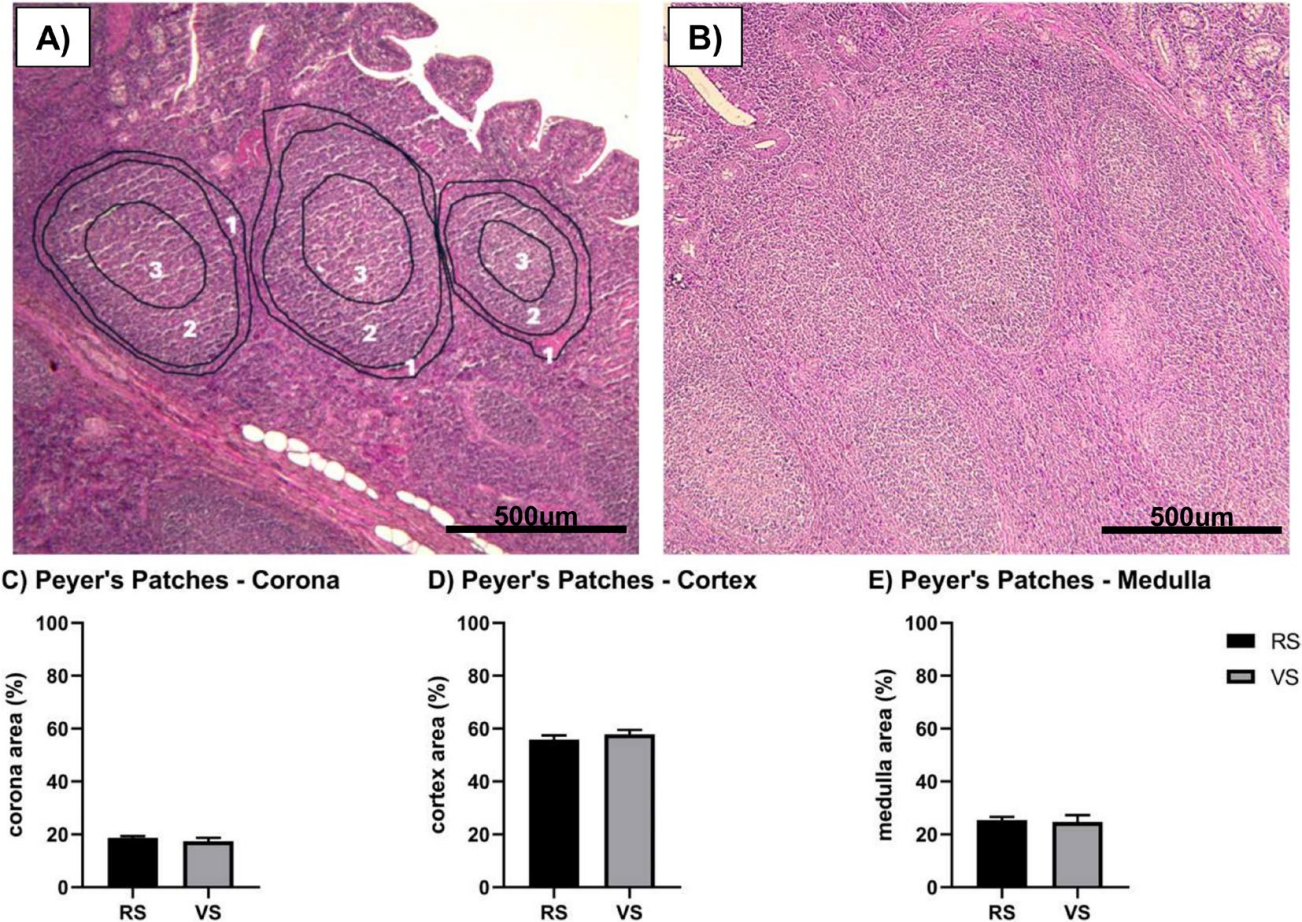
Peyer's Patches analysis. (A) Representative figure of H&E staining of RS ileum samples, where 1 is the corona area, 2 is the cortex area, and 3 is the medulla area. (B) Representative figure of H&E staining of VS ileum samples; (C) Percentage of the corona area in the ileum of animals from farms RS and VS. (D) Percentage of the cortex area in the ileum of animals from farms RS, and VS. (E) Percentage of medulla area in the ileum of animals from farms RS, and VS. *N* = 6 animals per experimental group. Results are expressed as mean ± SEM. Scale bar: 500 μm.

As in Figure 3A,B, the Peyer's patches were visible in all the samples, located in the ileum submucosa of RS and VS farms, respectively. Regarding the three areas analyzed, no significant differences were observed between the animals from the two farms (Figure 3C–E).

#### 3.3.2 Histochemistry

**FIGURE 4:**
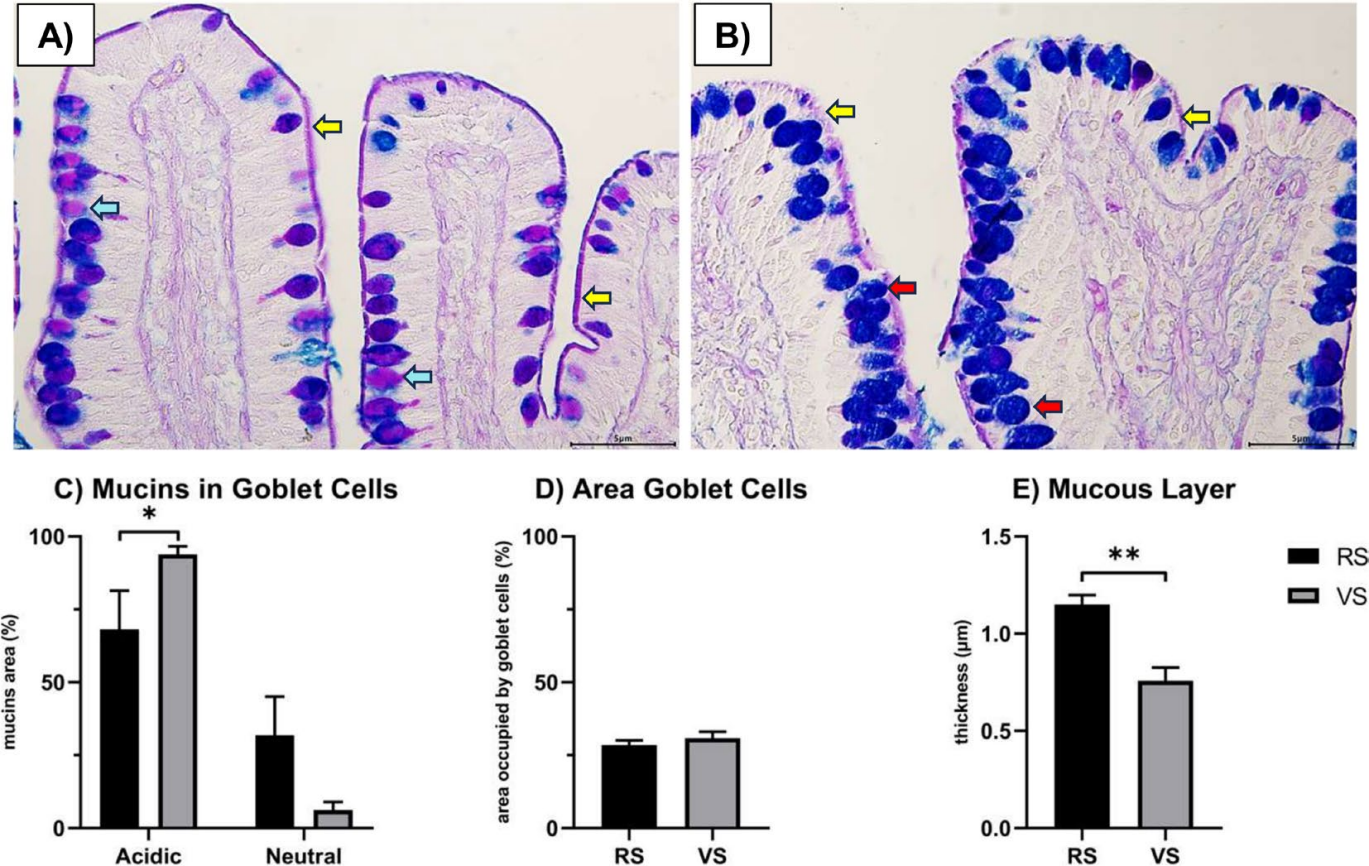
Goblet cells analysis. (A) Representative figure of AB/PAS staining of RS ileum samples. (B) Representative figure of AB/PAS staining of VS ileum samples. (C) Percentage of acidic and neutral mucins. (D) Percentage of area occupied by goblet cells in the epithelium. (E) Mucous layer thickness. Light blue arrow: Neutral goblet cell; yellow arrow: Mucous layer; red arrow: Acidic goblet cells. *N* = 6 animals per experimental group. Results are expressed as mean ± SEM. *p < 0.05; **p < 0.01. Scale bar: 5 μm.

The AB/PAS staining showed that intestinal goblet cells of the ileum contained neutral and acidic glycoconjugates in the animals of both farms (Figure 4A, RS farm, and 4B, VS farm). As for the type of goblet cells, in the animals from the RS farm, there was a significant reduction (*p* < 0.05, Figure 4C) in the number of acidic cells in favor of neutral ones compared to the animals from the VS farm. Nonetheless, the percentage of area occupied by goblet cells in the epithelium showed no significant differences between the two experimental groups (*p* > 0.05, Figure 4D). The mucous layer thickness was higher in animals from the RS farm than in animals from the VS farm (*p* < 0.01, Figure 4E).

#### 3.3.3 Double Immunofluorescence

**FIGURE 5:**
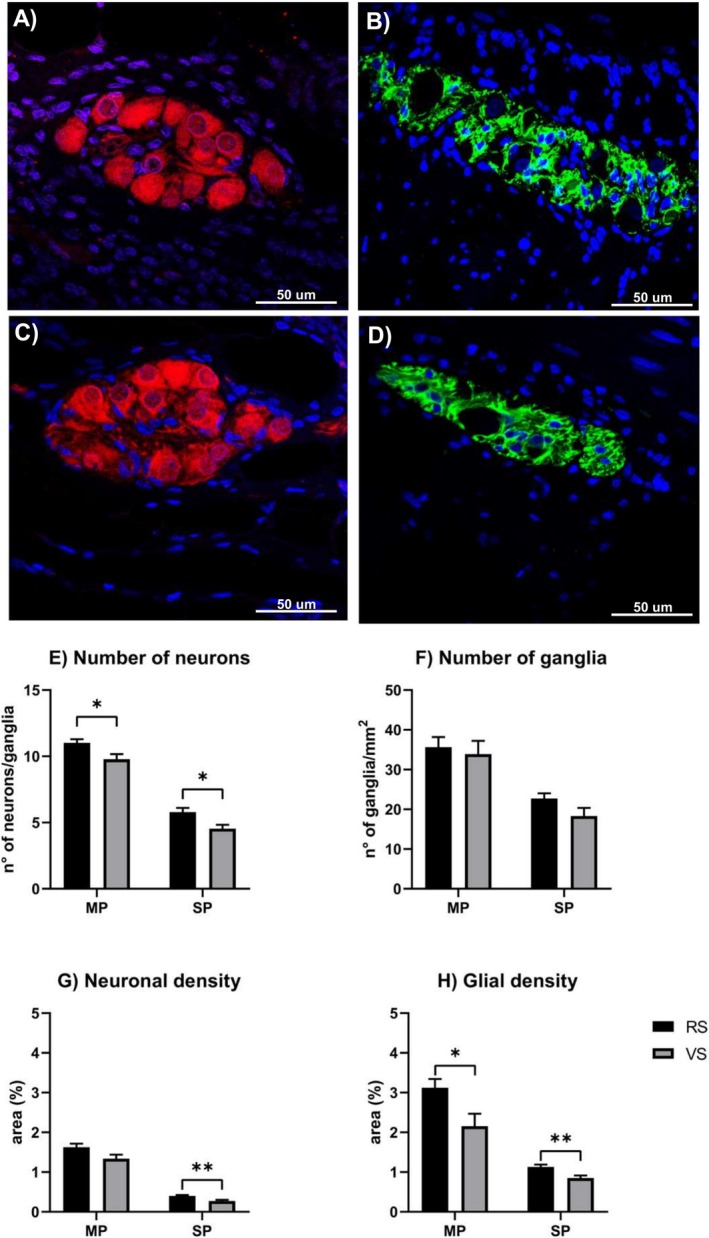
Immunofluorescence analysis in the ileum of animals from the RS and the VS farms. (A) Representative figure of neuron immunofluorescence with anti‐PGP9.5 antibody in SP; (B) Representative figure of glia immunofluorescence with anti‐GFAP antibody in SP. (C) Representative figure of neuron immunofluorescence with anti‐PGP9.5 antibody in MP; (D) Representative figure of glia immunofluorescence with anti‐GFAP antibody in MP. (E) Number of neurons in MP and SP; (F) Number of ganglia in the MP and SP; (G) Neuronal density in MP and SP; (H) Glial density in MP and SP. PGP9.5, red immunofluorescence, GFAP green immunofluorescence, nuclei blue. *N* = 6 animals per experimental group. *p < 0.05; **p < 0.01. Scale bar: 50 μm.

Double immunofluorescence revealed the presence of both neuronal cell bodies in SP and MP (PGP9.5, Figure 5A,C) as well as the enteric glial cells (GFAP, Figure 5B,D), which were detected as small cell bodies and their cytoplasmic processes in ganglia. The number of neurons both in the MP and in the SP was significantly higher in animals from the RS farm (*p* < 0.05 in Figure 5E‐MP and *p* < 0.05 in Figure 5E‐SP, respectively). The number of ganglia per mm^2^ was not different in the two groups, neither for the MP nor for the SP (*p* > 0.05 in Figure 5F‐MP and *p* > 0.05 in Figure 5F‐SP, respectively). Compared to the area of the entire section, the area occupied by neuronal cells in the MP showed no differences (*p* > 0.05 in Figure 5G‐MP), while those in the SP were higher in the RS (*p* < 0.01 in Figure 5G‐SP). The area of glial cells was greater in the animals from the RS farm in both the myenteric and submucosal ganglia (*p* < 0.05 in Figure 5H‐MP and *p* < 0.01 in Figure 5H‐SP, respectively).

As for the ratio between neuronal and glial density, no differences were found, neither in MP (RS: 2.12 ± 0.15 vs VS: 2.68 ± 0.31; *p* = 0.14) nor in SP (RS: 1.43 ± 0.06 vs VS: 1.28 ± 0.12; *p* = 0.27).

### 3.4 Neurotransmitters Gene Expression

**FIGURE 6:**
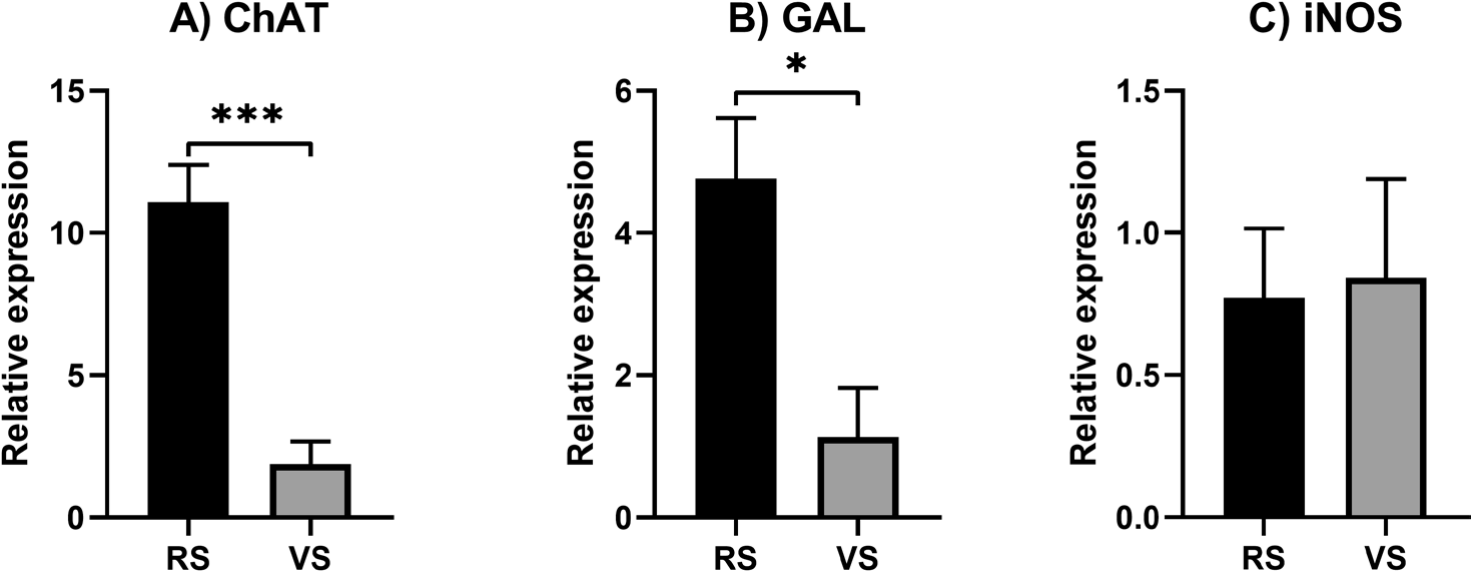
Gene expression of (A) ChAT, (B) GAL, and (C) iNOS in the ileum of animals from farm RS and farm VS. *N* = 5 per experimental group. *p < 0.05; ***p < 0.001.

*ChAT* and *GAL* genes were overexpressed in animals from the RS farm (*p* < 0.001 in Figure 6A, *p* < 0.05 in Figure 6B, respectively), while no significant differences were observed in the expression of *iNOS* (Figure 6C).

## 4 Discussion

NH_3_ pollution generated by pig farms can originate from animals and the decomposition of piggery waste manure. This gaseous effluent leads to many environmental problems, affecting the atmosphere, the neighborhood, and the health of animals and pig keepers [44]. This work studied chronic NH_3_ exposure on intestinal health and the plasticity of the ENS in pigs. The results also shed light on farm management and workplace safety. Indeed, the two farms studied adhered to BAT, but the results highlighted critical gaps in current environmental contaminant mitigation strategies, revealing significant differences in NH_3_ levels and their consequent effects on ENS response.

In the two farms, results put in evidence a higher ammonia concentration in the RS facility. This can be explained by the wrong management in manure removal, since the farmer recirculated digestates, richer in ammonia than manure, produced by the anaerobic digester plant, vanishing the mitigating potential of the BAT structure [45]. In the RS farm, ammonia ranged from 10 to 15 ppm, and from 3 to 5 ppm in VS in farrowing. In the fattening units, ammonia values ranged from 3 to 10 ppm in RS and from non‐detectable values to 12 ppm in VS. However, these mean values may be higher in comparison with 24 h measurements, since ammonia sampling was performed from 9 a.m. to 5 p.m., when animal activity and ventilation rate are greatest: authors reported lower ammonia concentrations in piggeries during the night, up to 30% [46], and 7% lower ammonia emission measured from 8 a.m. to 6 p.m. [47]. This variation in ammonia level is linked to the animal activity [3] during the day, and to the excretory behavior of the pigs [48], which also shows diurnal different patterns in the daytime [49].

The study confirmed that manure management systems greatly influence ambient NH_3_ levels. The VS farm, which used a manure vacuum system, consistently recorded lower NH_3_ concentrations than the RS farm, which used a recirculation system [50]. Although recorded levels in the fattening phases were below the regulatory threshold of 7 ppm [51, 52, 53], chronic exposure to high ammonia levels (mean value of 12 ppm) during farrowing seemed be to elicit adverse effects on gut health and ENS functionality. The intestine reacts to pathogens through the activation of the intestinal immune system, which, if it does not respond adequately, can lead to pathologies [54]. Borbet et al. [55] observed that in young mice, treatments with antibiotics altered the intestinal microbiome and the development of the GALT, affecting the germinal center of B cells of Peyer's patches and, consequently, altering the host immune response. In our study, no difference was observed between the two experimental groups in the three areas of the PPs, thus revealing that GALT has not been activated as a consequence of the high levels of NH_3_. While the absence of GALT activation suggests immune system modulation, this hypothesis warrants further investigation to clarify its implications [56, 57, 58, 59, 60]. The mucosal changes observed in farm RS pigs indicate NH_3_'s direct influence on intestinal defenses. During the prenatal, neonatal, and weaning periods, the porcine gastrointestinal tract undergoes significant structural and functional modifications, which are crucial for maintaining gut health and resilience against environmental stressors [61]. A reduction in acid glycoconjugates, critical for bacterial resistance, and an increased thickness of the mucus layer were evident in these animals, as already observed in pigs in response to a wide range of luminal insults, including alterations of the normal microbiota [62]. We did not observe any abnormalities of the intestinal microflora in either of the two farms, but the higher incidence of diarrhea observed in RS animals is possibly due to a compensatory defense mechanism given by the increase in mucus thickness and peristalsis [59, 63, 64, 65, 66]. These results suggest that such adaptive mechanisms of the intestinal mucosa can compromise its functionality despite the attempt to protect it from luminal insults, as reviewed by Herath et al. [67]. The detrimental effects of NH_3_ on respiratory and cardiovascular health are well‐documented: NH_3_ can mediate oxidative stress and inflammation in the lung tissue of pigs and the heart of chickens ([68, 69] respectively) and induces apoptosis in the liver of chickens [70]. However, the effects of environmental NH_3_ on gut health to date are scarce. In a recent study, Li et al. [12] assessed the effect of excessive NH_3_ exposure in fattening pigs on jejunal health and observed an increase in the levels of inflammatory markers as well as a beta diversity of intestinal microflora. The same authors observed that excessive NH_3_ can induce oxidative stress in the jejunum and cause histological injury [71]. In pigs, it was also described induction of dysbiotic microbiota in the hindgut following high NH_3_ exposure [72]. In our study, animals from the RS farm had a worse fecal score than VS but with similar bacterial populations, indicating that the animals did not have loose stools due to ongoing intestinal pathologies. We observed that there was an increase in the number of neurons, as well as an increase in the density of glial cells. Moreover, in this study, the higher glial density in both plexuses observed in RS animals supports a glial response to chronic NH3 exposure, potentially indicative of inflammation as reviewed by Gulbransen and Brown [73]. To our knowledge, there are no studies quantifying this ratio in pigs, making species‐specific comparisons challenging. However, our findings of increased glial density in RS animals align with the hypothesis of inflammation, consistent with observations by Leven et al. [74]. The neurons/glial cells ratio varies significantly between species: in mice, it is 1:1 in myenteric ganglia (MP) and 2:1 in submucosal ganglia (SP); in guinea pigs and rabbits, glia outnumber neurons 2:1 in MP but are nearly equal in SP. Larger animals, such as sheep, show a greater proportion of glia, with four times as many glial cells in MP and 1.5 times in SP [73]. Humans have the highest known ratio, with glia outnumbering neurons up to 7:1 in MP and 2:1 in SP [73]. These data underline the species‐specific variability in glial density, which increases with body size, reinforcing the importance of contextualizing findings within each species' unique physiology [73].

These findings align with existing conditions of neuroinflammation, such as Inflammatory Bowel Disease (IBD) and Crohn's Disease (CD), where ENS changes are implicated in disrupted gut homeostasis, as reviewed by Magalhães and Castelucci [75]. In patients with CD, defects in enteric glial networks have been observed [76, 77], frequently associated with neuronal damage, loss of neural connections, and neuroinflammation [78]. Not only neurons, but also EGCs may play a more active role in the control of gut function [79]. In transgenic mouse models, where EGCs are selectively ablated, the loss of glia resulted in intestinal inflammation and disruption of the epithelial barrier. Enteric glia are activated specifically by antigens and may actively contribute to inflammatory pathology via antigen presentation. Thus, enteric glia may serve as a link between the nervous and immune systems of the gut and may also have an important role in maintaining intestinal homeostasis [80]. In 2017, Fried et al. [81] observed that NH_3_ affected the enteric neuromuscular transmission in mice, pigs, and humans in an ex vivo study, by increasing intestinal motility through mechanisms involving the enteric glial cells. For this reason, our results suggest an activation of the glial cells due to inflammatory stimuli caused by NH_3_. These structural changes observed in the ENS appear to also be reflected at the molecular level, as demonstrated by the gene expression results. How the neuro‐immune interaction works at the intestinal level is not yet clear; the few studies in the literature date back to recent years [82]. We observed neuroplasticity with an upregulation in the expression of the *ChAT* gene; it is, therefore, plausible that the diarrhea observed in these animals was linked to an overactivation of the *ChAT* gene with the consequent increase in peristalsis. Our results revealed a greater expression of the *GAL* gene in the animals from the RS farm, and considering that at the gastric level, *GAL* has an inhibitory effect on acid secretion [83], hence it may be responsible for the decrease in acidic goblet cells in favor of neutral ones. Furthermore, *GAL* shows a modulatory effect on intestinal motility directly by acting on the smooth muscle cells of the gut or indirectly by neuromodulation and stimulating the synthesis of other factors [84, 85] and this could justify the presence of diarrhea in animals from farm RS. Unlike neuronal *NOS*, *iNOS* is expressed only under stimulation, usually by pro‐inflammatory cytokines [30]. The activation of *iNOS* can occur depending on the inducer; different pathways can be activated. *iNOS* gene activation depends on both cell type and species [86] and—among other cells—in muscle cells [87], glial cells [88] and neurons [89]. We observed a significant increase in glial activity in the RS group (detected by RT‐PCR), and no changes in *iNOS* expression, which remained similar between the two groups. This discrepancy could be explained by the rapid response of the glia. In support of this hypothesis, Rosenbaum et al. [90] showed that LPS‐induced intestinal inflammation causes immediate activation of glia in rats, a result that is consistent with what was observed in glia from RS pigs, but further research is needed to better understand this relationship and its significance. Indeed, enteric glia contribute to oxidative stress through the local production of nitric oxide during inflammation [91].

## 5 Conclusions

The general portrait from this study reveals that although the RS farm has been considered a BAT system for reconstructed confinements, the manure recirculation still developed levels of NH_3_ coming from the digesta, such that chronic exposure can create harmful events on the intestine. The higher levels of NH_3_ appeared to activate both mucosal and nervous responses in the ileum of pigs (chronic exposure). Results may have important implications not only for animal health but also for human occupational health/safety. Farm workers exposed to levels of NH_3_ like those observed in pigs can face risks of respiratory, cardiovascular, and now we know also intestinal neuroplasticity. Pigs, as animal models for humans, offer valuable insights into the complex interaction between environmental pollutants and gut health, paving the way for innovative solutions to mitigate these risks. Given the impact of chronic NH_3_ exposure on gut health, farm environments must prioritize ventilation improvements and explore alternative manure management technologies to mitigate these risks. Further investigations regarding the immunological and systemic aspects will be necessary, but the pig could represent a good model to evaluate the effect of environmental pollutants on intestinal health.

## Author Contributions

Substantial contributions to the conception or design of the work, or the acquisition, analysis, or interpretation of data for the work: V.R.H.M., M.S., K.P., G.M., C.C., M.M., L.A., L.M., C.B., E.B., A.C., S.C.B.M., A.D.G. Drafting the work or revising it critically for important intellectual content: V.R.H.M., M.S., K.P., G.M., C.C., M.P., L.A., L.M., C.B., E.B., A.C., S.C.B.M, A.D.G. Final approval of the version to be published: V.R.H.M., M.S., K.P., G.M., C.C., M.P., L.A., L.M., C.B., E.B., A.C., S.C.B.M., A.D.G. Agreement to be accountable for all aspects of the work in ensuring that questions related to the accuracy or integrity of any part of the work are appropriately investigated and resolved: V.R.H.M., M.S., K.P., G.M., C.C., M.P., L.A., L.M., C.B., E.B., A.C., S.C.B.M., A.D.G.

## Conflicts of Interest

The authors declare no conflicts of interest.

## Data Availability

The data that support the findings of this study are available from the corresponding author upon reasonable request.
